# Low autochtonous urban malaria in Antananarivo (Madagascar)

**DOI:** 10.1186/1475-2875-5-27

**Published:** 2006-03-31

**Authors:** Léon Paul Rabarijaona, Frédéric Ariey, Robert Matra, Sylvie Cot, Andrianavalona Lucie Raharimalala, Louise Henriette Ranaivo, Jacques Le Bras, Vincent Robert, Milijaona Randrianarivelojosia

**Affiliations:** 1 Institut Pasteur de Madagascar, BP 1274 Antananarivo-101, Madagascar; 2 Institut Santé et Développement, Université Pierre et Marie Curie, Université Pierre et Marie Curie Paris VI, 12 rue Cuvier, 75005 PARIS, France; 3 Service de lutte contre le paludisme, Ministère de la Santé et du Planning Familial, BP 460, Soarano, Analakely, Antananarivo-101, Madagascar; 4 IRD UR77/MNHN USM504, 61 rue Buffon, 75231 Paris cedex 05, France; 5 Institut Pasteur de Phnom Penh, Cambodge

## Abstract

**Background:**

The study of urban malaria is an area undergoing rapid expansion, after many years of neglect. The problem of over-diagnosis of malaria, especially in low transmission settings including urban areas, is also receiving deserved attention. The primary objective of the present study was to assess the frequency of malaria among febrile outpatients seen in private and public primary care facilities of Antananarivo. The second aim was to determine, among the diagnosed malaria cases, the contribution of autochthonous urban malaria.

**Methods:**

Two cross-sectional surveys in 43 health centres in Antananarivo in February 2003 (rainy season) and in July 2003 (dry season) were conducted. Consenting clinically suspected malaria patients with fever or history of fever in the past 48 hours were included. Malaria rapid diagnostic tests and microscopy were used to diagnose malaria. Basic information was collected from patients to try to identify the origin of the infection: autochthonous or introduced.

**Results:**

In February, among 771 patients, 15 (1.9%) positive cases were detected. Three malaria parasites were implicated: *Plasmodium. falciparum *(n = 12), *Plasmodium vivax *(n = 2) and *Plasmodium. ovale *(n = 1). Only two cases, both *P. falciparum*, were likely to have been autochthonous (0.26%). In July, among 739 blood smears examined, 11 (1.5%) were positive: *P. falciparum *(n = 9) and *P. vivax *(n = 2). Three cases of *P. falciparum *malaria were considered to be of local origin (0.4%).

**Conclusion:**

This study demonstrates that malaria cases among febrile episodes are low in Antananarivo and autochthonous malaria cases exist but are rare.

## Background

There is a hundred year-old history of control of malaria in African urban enviroment [1]. However, increasing attention is being devoted to this issue as a result of the rapid growth of cities and the fact that by 2025, more than 50% of the people in Africa are expected to be living in urban centres [2]. Despite its broad usage, the word 'urban' has an amazingly varied set of definitions. Most studies investigating the health consequences of urbanization employ ecological and cultural context comparisons of individuals living in urban versus rural environments [3-7]. There is no simple association between urbanicity and health [6], but the rapid increase of the world's urban population has major implications for the transmission and epidemiology of malaria and other vector-borne diseases [8,9].

In most countries of sub-Saharan Africa, malaria transmission is generally intense in rural areas. In contrast, malaria transmission occurs at significantly lower levels in urban areas, where population density is high, and where the number of mosquito breeding sites is reduced [10,11]. Few data are available to prioritize strategies for malaria control. Many questions remain for public health specialists hoping to regain control over malaria in urban environments [11-13].

In the central highlands of Madagascar, at the end of the French colonial era in the 1950s, malaria and its main vector *Anopheles funestus *has been suppressed to near or complete eradication. This was achieved by spraying Dichlorodiphenyltrichloroethane (DDT) mixed with gamma-hexachlorocyclohexane (γHCH) in over a million houses and mass chemoprophylaxis of schoolchildren with chloroquine given 3 times per week in 2,375 treatment centres [14]. As a result, the percentage of positive blood slides in Antananarivo was reduced from 25–35% in the late 1940s to 0.18% (6 positive among 3,348 slides from children between two and 10 years of age) in 1955. *An. funestus*, which had been abundant in highland houses in the 1940s, could not be found in the area in 1955 [14]. Some spraying continued and there were centres at which chloroquine was available until the late 1970s, when this intervention was, regrettably, abandoned [15]. *An. funestus *crept back into the area between 1976 and the 1980s. A sporozoite positive individual was found in 1987 and a plea was made for urgent introduction of control measures [16]. This plea was not heeded in time and an explosive epidemic hit the highland region in the late 1980s, the prevalence of parasitaemia returned to the levels seen in the 1940s [17-19] and tens of thousands died amongst the non-immune population [20,21].

In 1988, to control the spread of the vector, a national control programme that was based on accessibility to chloroquine and DDT indoor spraying aimed at the foci where the most malaria cases were found. From 1993 to 1998, the programme was extended to all 27 districts of the central highlands, including all areas affected by malaria. The main targets of the control programme were the villages located in altitudes of 1,000–1,500 m, representing the majority of the human settlements in the central highlands. Five malaria vector control campaigns, the so-called "Operation de Pulvérisation Intra-Domiciliaire" (OPID) programmeme, were funded by the World Bank. The goal was to protect a population of 2.3 million inhabitants per year.

Five cycles of residual indoor treatment with DDT in the central highlands of Madagascar have resulted in low levels of malaria transmission in the study area. The low prevalence of the infection and the rarity of the main vector, *An. funestus*, observed at the end of the OPID programme were the basis for suspending the antivector interventions in 1998–1999. Instead, more targeted interventions, the "Campagne d'Aspersion Intra-Domiciliaire" (CAID), was introduced, which still uses 2 g DDT/m2. These latter interventions have consolidated the impact on the vector but have not succeeded in stopping transmission. Certain cities as Antananarivo are no longer included in the CAID [22,23].

There is now full control of the epidemics and a considerable decrease in malaria transmission in the Highlands. As a result, febrile episodes are now often considered as malaria attacks based only on a clinical diagnosis, although the real contribution of malaria to febrile episodes remains unclear.

Two cross-surveys in February 2003 (rainy season) and in June 2003 (dry and cold season) in primary health centres in Antananarivo were conducted. The principal aim was to detect malaria attacks during febrile episodes in Antananarivo and to determine if there is local transmission in Antananarivo. The usefulness of malaria Rapid Diagnosis Test (RDT) was also assessed for fever cases management in heath care centres.

## Methods

### Study site and population

Antananarivo is the capital of Madagascar, has a population of about 1.5 million and is located on the central highlands. The region has two seasons: a hot and rainy season from October to April and a cold and dry season from May to September. The average temperature range is 13.8°C to 23.8°C, and the average annual rainfall is 1365.3 mm. Antananarivo Renivohitra has an area of 80 km^2 ^(Commune Urbaine/CUA d'Antananarivo), although most of that area is not currently urbanized. Antananarivo consists of administrative, commercial, industrial and residential areas, with patches of agricultural land that are mostly rice fields. The town was thought to be a malaria transmission-free zone [19,22], but it suffered devastating malaria epidemics between 1980 and 1990 [19]. It is thought that intensive indoor spraying of DDT stopped the epidemics in the town [22]. After four years of spraying, the *An. funestus *mosquito population progressively disappeared and malaria transmission decreased [23].

### Dispensaries and patients

Two surveys were conducted: the first during February and March 2003 (rainy season) and the second in June and July 2003 (cold and dry season). Any patient having an axiliary temperature >37.5°C or with suspected malaria according to the consulting physician was included in the study.

**Figure 1:**
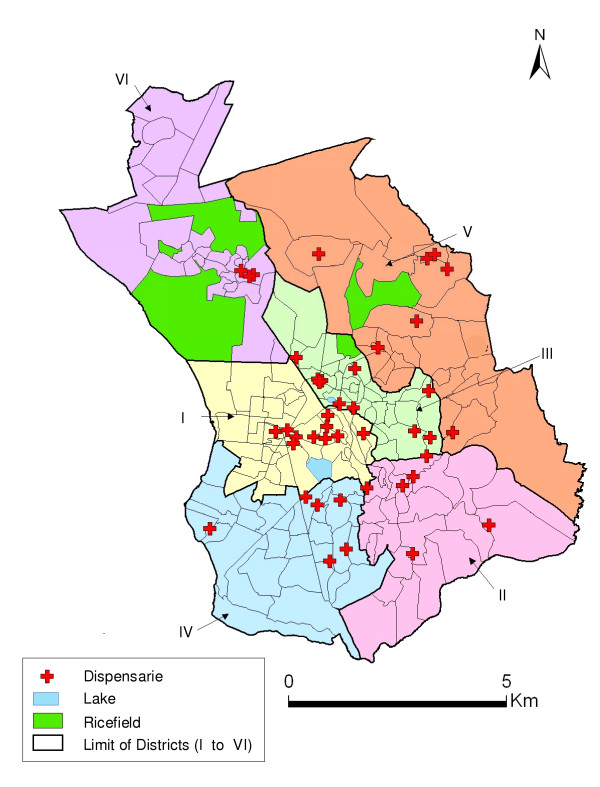
Six administrative districts of Antananarivo and dispensaries.

The survey was conducted in 43 public and private dispensaries distributed in the six administrative districts of Antananarivo Renivohitra. After declarations made in January 2002, dispensaries declaring more than two cases of fever per day were retained in the study. The locations of the participating dispensaries were mapped using a hand-held Global Positioning System (GPS) receiver (Figure 1). Each dispensary was visited during two days during their busiest periods.

**Parasitological surveys **were performed using two methods. Firstly, malaria diagnosis by microscopy. Thick and thin blood films were stained with Giemsa. The number of asexual stages of *Plasmodium falciparum *was counted against white blood cells in a same oil immersion field. The density of parasites was estimated using 8,000 white blood cells/μl of blood as a standard in the thick smear. Parasite counts were taken by a trained and experienced laboratory technician. Malaria RDTs were performed in all outpatients included in the study. Two types of RDTs were used: MakroMED^® ^(histidine-rich protein-II, Pf HRP-II, Makro Medical Pty. Ltd., Johannesburg, Republic of South Africa) during the February survey and OptiMAL-IT^® ^(DiaMed, under license from Flow Inc., Portland, Oreg., 1785 Cressier sur Morat, Switzerland) during the July survey. All blood samples were collected after informed consent from the patients. A completed questionnaire was obtained from each patient.

### Clinical diagnosis and treatment

The RDT results were not made available to the attending physician and were not used as a guide to treatment. Routinely, the physician made a clinical diagnosis (without biological diagnosis) and decided on treatment for the patient. The RDT result was only then given to the physician, on request.

### Survey of index cases of presumed indigenous malaria

In February-March, surveying the areas around the index cases of malaria presumed of local origin completed the study. Parasitological and entomological surveys were carried out.

#### Entomological monitoring and sporozoïtes detection

Adult mosquitoes were collected. The first method used pyrethrum space spray collections in the bedrooms of the two index cases and in the surrounding houses (32 rooms). The second method used human landing catches of adult mosquitoes collected twice, from February 26^th ^to the second of March and from April 15^th ^to April 18^th^, in the index case areas: Manakambahiny and Fenomanana. At each site, two outdoor and two indoor bait collectors were used. Collection was carried out from 18:00 to 06:00 hours, with a change of teams at midnight. The anophelines were identified in laboratory using the Grjebine morphologic identification keys. After identification, the anophelines were dissected and the heads and thoraces were tested by ELISA for *P. falciparum *circumsporozoite protein (CSP) as described by Burkot *et al*. [24] and modified by Wirtz *et al*. [25]. The ovaries of anopheline vectors were examined for parity using the Detinova technique [26]. The biting rates for the collected anophelines were calculated.

#### Malaria of local origin

A malaria case (non-corrected axiliary temperature ≥37.5°C and asexual parasites in the blood film) was considered as having a local origin for patients who declared no travel out of urban Antananarivo during the previous four weeks. Active case detection was conducted among an index case family co-habitation and neighbour by RDT and thick and thin blood films.

#### Molecular analysis of autochthonous *P. falciparum*

The genetic diversity of *P. falciparum *isolates was studied, according to patients and their geographical location, using *msp2 *encoding highly polymorphic loci from merozoite surface protein genes. DNA was extracted and amplified by polymerase chain reaction (PCR) using the following specific primers of *P falciparum*: *msp2*-3' et *msp2*-5'. *Msp2 *was genotyped by using nested PCR; specific fluorescent primers were used in the nested PCR for assignment to the 3D7- or FC27-type *msp2 *family.

### Data analysis

Data was entered, processed and analysed using EPI INFO (CDC Atlanta, version 6.04). Frequencies were calculated and the χ^2 ^test was used for differences between proportions. A value of p < 0.05 was considered significant.

### Ethical clearance

The study was approved by the Ministry of Health and the National Ethics Committee, Madagascar. Permission and informed consent were obtained from the Antananarivo district and the outpatients of the study.

## Results

During the study, 771 of 5,428 outpatients were included in the 43 dispensaries during March/February for 739 of 5,314 outpatients in June/July. The populations characteristics (age, sex) were comparables between the two seasons (Table 1).

**Table 1:** Study population characteristics. February and June 2003, Antananarivo

		February 2003 n = 771	June 2003 n = 739	*p*
Age	< 5 years	50%	51.5%	NS
	= 5 years	50%	48.5%	
Gender	Masculin	51.1%	47.2	NS
	Féminin	48.8%	52.8	
Temperature	> 37.35°C	75.7%	79.4	NS
	≤37.5°C	24.3%	20.6	

### Malaria prevalence

In February-March, of the 771 blood films examined, 15 (1.9%) were found to be infected: 12 *P. falciparum*, two *Plasmodium vivax *and one *Plasmodium ovale*. The average parasitaemia was 14,565 parasites/μl (minimum 47/μl, maximum 54,250/μl). Two *P*. *falciparum *malaria cases among the 771 subjects could be considered of local origin. MakroMed^® ^test correctly detected the 12 *P. falciparum *cases.

**Figure 2:**
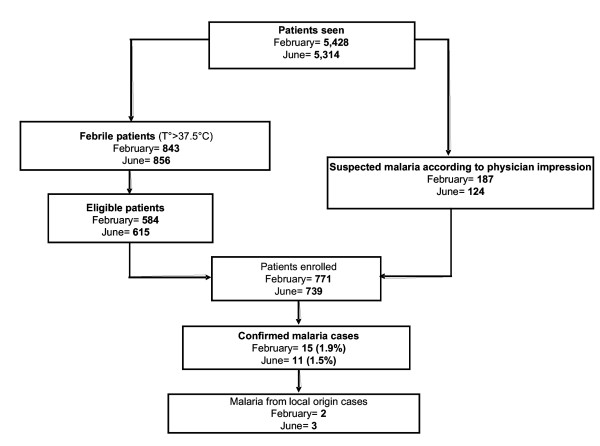
Study patients profile. February and June 2003. Antananarivo.

In June-July, of the 739 blood films examined, 11 (1.5%) were found to be infected: nine *P. falciparum *and two *P. vivax*. The average parasitaemia was 9,762/μl (minimum 16/μl, maximum 98,500/μl). Three *P. falciparum *malaria cases among the 739 subjects (0.4%) could be considered of local origin (Figure 2).

The suspected indigenous malaria cases were located in the same neighbourhood for the two seasons. Of the nine patients having falciparum parasitaemia as determined by microscopy, six patients had a positive *p*LDH antigen detection dipstick test. Of the two patients having presence of *P. vivax *in the microscopic samples, the OptiMAL-IT^® ^test correctly detected the presence of malaria in one of these samples.

### Clinical diagnosis and treatment

With regard to seasonal patterns, presumptive malaria rates varied from 59.2% (55.8–62.7) of all diagnoses in February to 32.9% (29.6–36.3) in July. Respiratory problems were diagnosed in 46.5% of the cases in July. However, the proportion of antimalarial drugs prescribed did not differ between the two seasons. Generally, there was a frequent prescription of an antimalarial and antibiotic together. In February, among 771 outpatients, 375 (48.7%) were treated with antimalarial drugs. Among these 375 subjects, 225 (60%) had both antimalarial drugs and antibiotics. In July, among 739 outpatients, 365 (49.4%) were treated with antimalarial drugs. Among these 365 subjects, 150 (41.1%) had both antimalarial drugs and antibiotics.

### Surveys of index cases of presumed indigenous malaria

**Figure 3:**
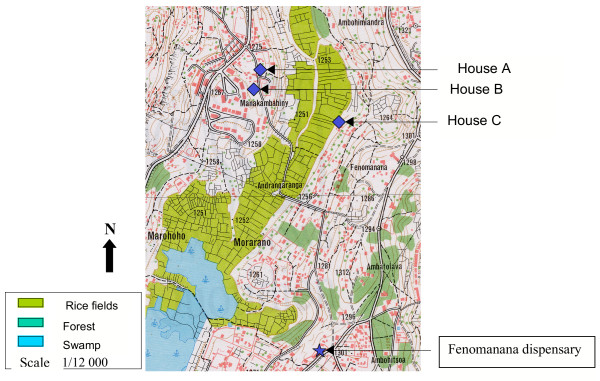
Autochtonous malaria cases location. February 2003, Antananarivo.

In February, in surveys made around the two index cases, five subjects were RDT positive. Thick and thin blood films confirmed the presence of *P. falciparum *for four subjects. They had not travelled out of urban Antananarivo during the previous four weeks. The two index cases and the four subjects are located in three houses (Table 2 and Figure 3).

**Table 2:** Index cases and secondary cases. February 2003, Antananarivo

Inclusion Number	Age	Sex	House identification	RDT	Thick and thin film	Parasitaemi a	Clinical manifestation
92 (index case)	39	F	B	+	+	53 750/μl	Fever, vomiting
93 (index case)	14	F	C	+	+	9 500/μl	Fever, asthenia
95	60	M	C	+	+	45 647/μl	Asymptomatic
91	12	F	A (neighbour of C)	+	+	2 040/μl	Fever, asthenia
94	11	M	A (neighbour of C)	+	+	2 920/μl	Fever
89	62	F	A (neighbour of C)	+	+	240/μl	Asymptomatic
90	21	F	A (neighbour of C)	+	-	0	Fever

The entomological survey detected three vectors: *Anopheles arabiensis, An. funestus and Anopheles mascarensis*. For the collection of adult anophelines, *An. arabiensis *was the most common species found during the two study seasons. Human-bait collection was the most successful method (Table 3).

**Table 3:** Adult *Anopheles *mosquitoes collected by different methods in Antananarivo. February and April 2003

**Species**	**Human landing catches**		**Pyrethrum spray catches**	**Total (%)**
	February	April		
*An. arabiensis*	194	142	19	355 (99.4)
*An. funestus*	1	0	0	1 (0.3)
*An. mascarensis*	0	1	0	1 (0.3)

Total	195	143	19	357

Of the 338 anophelines (*An. arabiensis*, *An. funestus*, and *An. mascarensis*) collected and examined by ELISA to detect circum sporozoite protein, none was found positive. The biting rates of *An. arabiensis*, the most common species, were relatively low. *An. arabiensis *manifested exophagic tendencies (Table 4). Parity rates were 57% in February-March and 53% in April.

**Table 4:** Daily biting rates of anophelines caught on human landing catches indoors and outdoors in Antananarivo. February and April 2003

**Species**	**February**	**April**
*An. arabiensis *indoors	1.33	0.96
*An. arabiensis *outdoors	6.83	4.66
*An. funestus *outdoors	0.04	0
*An. mascarensis *outdoors	0	0.04

**Figure 4:**
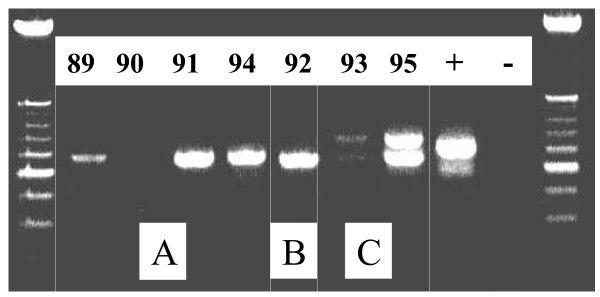
PCR amplification of the *msp2 P. falciparum *for seven infected subjects. February 2003, Antananarivo (A, B, C: House identification/89, 90, 91, 92, 93, 94, 95: Inclusion number)

PCR amplification of the *msp2 P. falciparum *for seven infected subjects (one subject was RDT positive but slide negative) showed polymorphism (Figure 4). Genetic analysis of *P. falciparum *strains allowed distinguishing three genotypes, aggregated by house. The three *P. falciparum *samples genotyped from the subjects 89, 91 and 94 (house A) showed an identical band with a size of 600 pb. The subject 92 (House B) showed a band of 550 pb size. The subjects 93 and 95 (House C) showed two identical bands of a size from approximately 600 pb and 650 pb. There was no amplification of DNA from the subject 90 who was RDT positive but slide negative. The nested PCR determined that all *P. falciparum *samples belonged to the 3D7 *msp2 *allelic family. Genotypes were also aggregated by house on the same mode as that obtained with the *msp2 *primers.

## Discussion

It was originally thought that urban areas do not support significant levels of malaria transmission. The concentration of human populations in small areas is normally accompanied by pollution and destruction of the clean water sources needed by the anopheline vectors of malaria. However, this is true for formal urban development but not for the informal development that occurs in sub-Saharan African countries and in Madagascar.

From the parasitological survey, 1.9% and 1.5% of the study population were found to be infected in February-March and in June-July, respectively. *P. falciparum *was the most common species followed by *P. vivax *and *P. ovale*. It is known that *P. falciparum *is the dominant species in Madagascar [14,17,27]. In the same epidemiological facies (highland malaria in an urban area), Woyessa and *al*. also showed a relatively low proportion of malaria cases in Akaki Town, Ethiopia. Among 2136 blood films, they found 3.7% malaria positive with 69% of them due to *P. vivax *and 31% due to *P. falciparum *[28]. In N'Djaména, Chad (urban malaria in Sahelian towns with highly seasonal malaria transmission), Othnigué and al. found that among 1,658 patients 30% were positive for *Plasmodium *(all *P. falciparum*)[29].

In the present study, there were very few *P. falciparum *malaria cases in both the low and high transmission seasons. The intensive epidemic control measures, such as treatment of confirmed cases and especially vector control activities, after the 1986 outbreaks are thought to have contributed to the low infection rate [23,30,31].

This study confirms that malaria cases in Antananarivo are mostly associated with travel to areas with high levels of malaria transmission, particularly the highlands fringe areas or the coastal regions of Madagascar. Nevertheless, autochthonous malaria transmission is suspected in some locations. The surveys around the two index cases seem to confirm this observation:

(i) Six subjects diagnosed positive, around the index cases, confirming that they had not travelled out of urban Antananarivo during the previous four weeks.

(ii) Anopheline vectors (a great majority of *An. arabiensis*) were captured by pyrethrum spray collections in the bedrooms of the two index cases and in the surrounding houses and by human landing catches of mosquitoes. The principal malaria vector on the Madagascar Highlands is *An. funestus*. More than 95% of its breeding sites are in the rice fields just before the harvest and then afterwards in the fallow lands. The vector peak and corresponding peak of malaria cases occurs between February and May, depending on the farming calendar. The second but less important vector, *An. arabiensis*, breeds in rice fields just after seeding when the surface water is sunlit. Although rice fields are the principal breeding site for this vector, it also breeds in rainwater pods [27]. From these findings, *An. arabiensis *may be the most important species involved in the transmission of malaria in Antananarivo, although it occurs in relatively small numbers compared to other areas [32]. The absence of sporozoïtes infection in this species may also be due to the small sample size of mosquitoes. Only, one *An. funestus *was aggressive for human in February-March. However, more detailed studies are required on bionomics of *An. arabiensis *and *An. funestus *to determine their exact role in malaria transmission in urban Antananarivo.

(iii) The six thick positive subjects were located in three houses near rice fields, an environment shown to be favourable for vector breeding [27,33].

(iv) Genetic analysis of *P. falciparum *strains allowed to distinguish three genotypes, aggregated by house.

Generally, malaria transmission is negatively associated with urbanization as shown in Kinshasa [34]. This is primarily due to overcrowding, to elimination of breeding sites due to construction, or to pollution of the breeding places resulting in lower vector densities in urban centres. However, in Antananarivo, autochthonous malaria transmission could also be maintained for short periods of time [22,27,33] in locations with rice fields. A low level of transmission may persist in these areas and may lead to further outbreaks of malaria in the future due to the presence of anopheline vectors. Thus, sustainable and integrated vector control measures and appropriate case management should play a crucial role in preventing further malaria epidemics. Moreover, studies need to be carried out to improve the epidemiological understanding of the cause and origin of the transmission.

The study demonstrates deficiencies in fever cases management in health centres, since many febrile episodes are wrongly identified as malaria, despite full control of the epidemics. Madagascar has now revised its national malaria treatment policy and is currently implementing a change from chloroquine to artemisinin combination treatment (ACT), particularly artesunate-amodiaquine. The low quality of malaria case management, including an over-prescription of antimalarials, mostly chloroquine but also quinine, highlights the problem of drug overuse and the associated risk of more rapid development of resistance. This study shows the importance of strengthening diagnosis and improving the treatment of indigenously transmitted malaria in areas of Antananarivo areas where diagnosis could be overlooked. The results show that RDTs would be useful and should be implemented in areas of transmission, particularly in circumstances that are not covered by traditional microscopic diagnosis of malaria.

## Authors' contributions

RLP is the principal investigator of the study and led the drafting of this manuscript; AF conceived the study and participated in its design and coordination; MR is investigator of the study, participated in the design of the study, supervised the acquisition of the data, helped analyse the data; CS, RAL and RLH, supervised the acquisition of the data, carried out laboratory analysis and helped analyse the data;

Le B J, RM and RV all conceived the study, took part in its development and revision.

All authors read and approved the final manuscript.
